# Systems-Mapping of Herbal Effects on Complex Diseases Using the Network-Perturbation Signatures

**DOI:** 10.3389/fphar.2018.01174

**Published:** 2018-10-18

**Authors:** Xuetong Chen, Chunli Zheng, Chun Wang, Zihu Guo, Shuo Gao, Zhangchi Ning, Chao Huang, Cheng Lu, Yingxue Fu, Daogang Guan, Aiping Lu, Yonghua Wang

**Affiliations:** ^1^Center of Bioinformatics, College of Life Science, Northwest A & F University, Yangling, China; ^2^School of Chinese Medicine, Institute of Integrated Bioinformedicine and Translational Science, Hong Kong Baptist University, Hong Kong, Hong Kong; ^3^Institute of Basic Theory for Chinese Medicine, China Academy of Chinese Medical Sciences, Beijing, China; ^4^Institute of Basic Research in Clinical Medicine, China Academy of Chinese Medical Sciences, Beijing, China

**Keywords:** systems pharmacology, herbal medicines, perturbation signatures, network pharmacology, personalized medicine, mathematical modeling

## Abstract

The herbs have proven to hold great potential to improve people's health and wellness during clinical practice over the past millennia. However, herbal medicine for the personalized treatment of disease is still under investigation owing to the complex multi-component interactions in herbs. To reveal the valuable insights for herbal synergistic therapy, we have chosen Traditional Chinese Medicine (TCM) as a case to illustrate the art and science behind the complicated multi-molecular, multi-genes interaction systems, and how the good practices of herbal combination therapy are applicable to personalized treatment. Here, we design system-wide interaction map strategy to provide a generic solution to establish the links between diseases and herbs based on comprehensive testing of molecular signatures in herb-disease pairs. Firstly, we integrated gene expression profiles from 189 diseases to characterize the disease-pathological feature. Then, we generated the perturbation signatures from the huge chemical informatics data and pharmacological data for each herb, which were represented the targets affected by the ingredients in the herb. So that we could assess the effects of herbs on the individual. Finally, we integrated the data of 189 diseases and 502 herbs, yielding the optimal herbal combinations for the diseases based on the strategy, and verifying the reliability of the strategy through the permutation testing and literature verification. Furthermore, we propose a novel formula as a candidate therapeutic drugs of rheumatoid arthritis and demonstrate its therapeutic mechanism through the systematic analysis of the influencing targets and biological processes. Overall, this computational method provides a systematic approach, which blended herbal medicine and omics data sets, allowing for the development of novel drug combinations for complex human diseases.

## Introduction

Common complex diseases are caused by a combination of heritable and environmental factors that affect the gene expression of individual (Kalf et al., 2014). In the past decades, drug design and development strongly focused on a limited number of targets considered crucial for disease (Sams-Dodd, 2005). Although enormous efforts have been made to obtain potent and specific drugs, the side effects caused by these drugs and the emergence of resistance to complex diseases are still not negligible (Lounkine et al., 2012; Holohan et al., 2013). By contrast, it becomes more and more evident that, for complex diseases like rheumatoid arthritis, an interference from multiple molecules and multiple targets is superior to classical “one-target-one-disease” approach regarding drug efficiency, side-effects and drug resistance (Sheng and Sun, 2011; Koeberle and Werz, 2014).

Traditional Chinese Medicine (TCM), whose therapeutic efficacy is based on the multi-target perturbation of a mixture of ingredients, offers new treatment opportunities by targeting signaling and metabolic pathways, and the mediation process of genetic central dogma (Efferth and Koch, 2011). Furthermore, the traditional herbal formulas have achieved tremendous achievements in clinical practice based on TCM clinical practice guidelines (e.g., “Jun-Chen-Zuo-Shi,” 
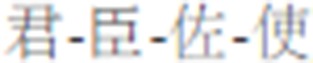
), which are the clinical summary of Chinese herbalists over the past millennia (He et al., 2015; Zhao et al, 2015). However, the synergistic therapeutic mechanism of the formulas is still blurred, and that resulting in the fatigued and weak of the formulas on modern complex diseases. Hence, the fundamental challenge that arises throughout TCM is the need to establish the relationship between diseases and the action of herb therapeutics.

**Figure 1:**
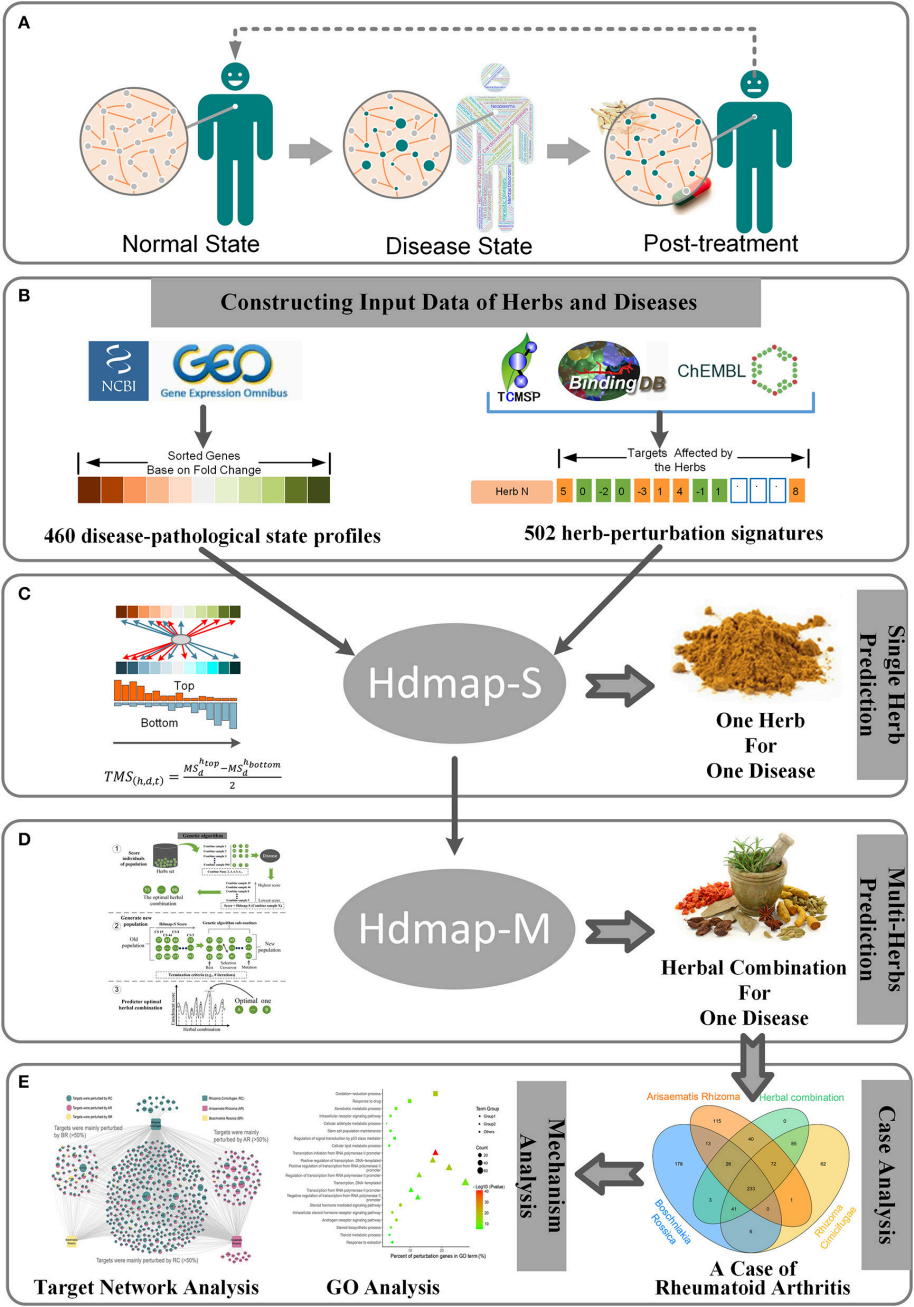
Overview and workflow used in this study. **(A)** Pathological changes in the human from the normal state to disease state and then to post-treatment. For each pathological state, the nodes in the biological network will change to characterize the current pathological state. **(B)** The preparation of the disease-pathological state profiles and herb-perturbation signatures for the algorithm in this study. **(C)** Description of the algorithm used to identify the optimal herb for a disease. **(D)** Description of the algorithm used to identify the optimal herbal combination for a disease. **(E)** A case study of the algorithm.

At present, the traditional paradigm of diagnostic and therapeutic methods in TCM is generally regarded as experiential, which is a result of the comprehensive analysis of a practitioner's clinical symptoms and signs based on the accumulated experience (Lu et al., 2012). Moreover, to ensure the treatment efficacy for the disease, a substantial portion of the clinical formulas contain multiple herbs. Although the combination of these herbs can be effective in treating diseases, the antagonistic effects or side effects of different ingredients in herbs could also lead to the decline of the patients' quality of life (Benzie and Wachtel-Galor, 2011; Shenefelt, 2011; Chan et al., 2012). Hence, identifying the optimal herb combination for disease opens a new approach to mitigate the burden and risks in clinical treatment, increase the understanding of the mechanisms of combination therapy, and offers new personalized and effective treatment solutions. And it is important to note that the rapid development of the analytical methods for diseases and herbs allows us to assess a disease's pathological state and herb's potential therapeutic qualities. Furthermore, a recent study has demonstrated that the persistent changes in gene expression direct shifts in the pathological states that including the occurrence, development, and healing of the disease (Figure 1A;Kleinjan and Van Heyningen, 2005). These conditions provide inspiration for us to solve this problem.

Here, we performed system-wide interaction map strategy between the expression signatures of human diseases and the perturbation signatures of the exogenous substances (e.g., the herbs or the herbal combination). Using this approach, our goal is to provide a generic solution to establish a link between diseases and herbs by attempting to describe the disease-pathological state in terms of genomic signatures, create a large database of perturbation signatures of herbs (Figure 1B), and develop pattern-matching tools to detect similarities among these signatures (Figures 1C,D). To address concerns about the application of our method, we present an instance that this strategy can confirm already known therapeutic uses of herbs and uncover new herb combination for diseases (Figure 1E). It is envisioned that this strategy will personalize medicine, not only for the diagnosis and treatment of common complex diseases (e.g., rheumatoid arthritis, type 2 diabetes, etc.) but also for the exploring of the synergistic therapeutic mechanism of herbs in the formula. And to our knowledge, systematic integration and analysis of herbal perturbation data and disease genome data have not been performed.

## Materials and methods

### Disease-pathological state profiles

To capture the transcriptional profiles in disease conditions, we traversed the transcriptional profile from GEO database (https://www.ncbi.nlm.nih.gov/geo/; Barrett et al., 2013) and calculated the differential expression genes by comparing disease-group samples and healthy control samples in the same experiment. During the analysis, the samples from different states, including disease-related status and health status, were identified for further analysis. Then, the RMA-normalized expression values of the samples were produced, and differential expression analysis was performed by the Bioconductor package (Gentleman et al., 2004; Robinson et al., 2010). To make cross-platform comparisons compatible, we standardized gene identifiers from probe identifiers of 45 sequencing platform to NCBI Human Gene Symbol identifiers, selecting individual probe with the minimum *p*-value. Finally, we represented the pathological state of diseases by a series of differentially expressed genes that were ranked by fold change. And we obtain 460 disease-related pathological state profiles (Supplementary Table 1).

### Herb-perturbation signatures

Herbal concoctions are a complex system, which contains many ingredients and plays a therapeutic role by hitting multiple biological targets involved in various pathogenesis (Huang et al., 2014). Hence, we represented the therapeutic effects of herbs as the herb-perturbation signatures, which are the targets that influenced by the ingredients in herbs. To create the perturbation signatures for each herb, we firstly obtained 502 herbs, 10,329 ingredients, and 31,288 herb-ingredient relationships from the Traditional Chinese Medicine Systems Pharmacology database (TCMSP), which is one of the TCM databases containing the most comprehensive molecular, target, and disease information in the world (Ru et al., 2014). To make the ingredients compatible with different platforms, we map the ingredients to the PubChem compound database to acquire the PubChem CID of each ingredient (Kim et al., 2015). Then, experimentally supported targets of each ingredient were extracted from two major public domain compound data repositories: the Binding Database projects (Liu et al., 2006) and ChEMBL database version 23 (Gaulton et al., 2011). The ChEMBL contains 2,275,906 small molecules and 12,091 protein targets. And the BindingDB contains 1,454,892 binding data, for 7,082 protein targets and 652,068 small molecules. These two databases are considered as the largest molecular-target databases supported by experimental data. To overcome the shortage of experimental data of the natural product molecules acting on the targets, a large-scale direct target prediction method, weighted ensemble similarity (WES) algorithm (Zheng et al., 2015) that recently developed by our group was performed to obtain the targets for each ingredient. To assess the pharmacological action of the ingredient and corresponding targets, a PreAM model developed earlier by our group was used to determine the relationship between ingredient and target in two types: activated (+1) or inhibited (−1) (Wang et al., 2015). Then, we construct the initial perturbation signatures for each ingredient. And the initial perturbation signatures of each herb were the superposition of the perturbation signatures of the ingredients in herb (Supplementary Figure 1A). During in the process of the superposition, we consider the influence of the content of ingredients on the perturbation signatures. If the content of herbal ingredients is not clear, we set its parameter to 1 in the calculation of the herbal perturbation signatures. If the ingredients' content is clear, the percentage of the ingredients is used as a parameter to calculate the perturbation signatures of the herb. Finally, we obtained the initial perturbation signatures of the herbs. Simultaneously, the initial perturbation signatures of the herbal combination were the superposition of the perturbation signatures of the herbs in herbal combination.

To further understand the influence of each ingredient/herb/herbal-combination on the whole biological network, the physical protein-protein interactions (PPI) with experimental support from several sources including regulatory interactions, binary interactions, literature-curated interactions, metabolic enzyme-coupled interactions, protein complexes, kinase network (kinase-substrate pairs), and signaling interactions, were obtained to establish a PPI network including 13,460 proteins and 141,296 interactions (Menche et al., 2015). And the correlation matrix of the PPI network was defined as *A*_*n* × *n*_:

$$\begin{array}{l} \alpha_{ij}=\{\begin{array}{c} 1\\ 0 \end{array}\;\left\{i,\;j\;|\;1\leq i\leq n,\;1\leq j\leq n\right\} \end{array}$$

where *n* is the total number of the proteins in PPI network and α_*ij*_ represents the interaction between the *i*-th protein and *j*-th protein: 1 (interaction) or 0 (no-interaction). Then, we converted the initial perturbation signatures of each ingredient into a “perturbation” matrix that each row vector represents the perturbation intensity of the protein by the ingredient/herb/herbal-combination. And the perturbation intensity means the degree of activation or inhibition that a protein affects by the ingredient/herb/herbal-combination. The row vectors for each ingredient/herb/herbal-combination was defined as follows:

$$\begin{array}{l} P\;=\left\{c_{1},\;c_{2},\;\ldots ,c_{i},\ldots ,c_{n}\right\} \end{array}$$

where *P* represents the initial perturbation signatures of the ingredient/herb/herbal-combination, *c*_*i*_ represents the perturbation intensity of the ingredient/herb/herbal-combination on the *i-*th protein where the initial value of an ingredient is +1 or −1 and the initial value of herb/herbal-combination is an integer. And the sign of *c*_*i*_ indicates different perturbation patterns: activated (+) or inhibited (–). Then, a thermal diffusion model was performed to explore the perturbation process of the ingredient/herb/herbal-combination through the PPI network so that we could obtain the final perturbation signatures for the ingredient/herb/herbal-combination (Supplementary Figure 1B). This model mainly consists of two steps. Here, the first-step of the thermal diffusion model was defined as follow:

$$\begin{array}{l} P^{step1}=\sum_{i=1}^{n}\alpha_{ij}\left(\frac{c_{i}}{D_{i}}\right)\\ P^{step1}=\{{c^{\prime }}_{1},\;{c^{\prime }}_{2},\;\ldots ,{c^{\prime }}_{i},\ldots ,{c^{\prime }}_{n}\} \end{array}$$

where *D*_*i*_ is the number of proteins that interact with the *i*-th protein in PPI network and ${c^{\prime }}_{i}$ represents the perturbation intensity of the ingredient/herb/herbal-combination on the *i-*th protein after first-step thermal diffusion. Then, the second-step of the thermal diffusion model was performed to the obtain the final perturbation signatures.

$$\begin{array}{l} P^{step2}=\sum_{i=1}^{n}\alpha_{ij}\left(\frac{{c^{\prime }}_{i}}{D_{i}}\right)\\ P^{step2}=\{{c^{\prime \prime }}_{1},\;{c^{\prime \prime }}_{2},\;\ldots ,{c^{\prime \prime }}_{i},\ldots ,{c^{\prime \prime }}_{n}\} \end{array}$$

where ${c^{\prime \prime }}_{i}$ represents the perturbation intensity of the ingredient/herb/herbal-combination on the *i-*th protein after second-step thermal diffusion and the *P*^*step*2^ is the final perturbation signatures of the ingredient/herb/herbal-combination. Finally, we obtained 502 herb-perturbation signatures including 13,486 perturbation protein ordered by perturbation intensity values (Supplementary Table 2).

### Prediction a single herb for the disease (HDmap-S)

**Figure 2:**
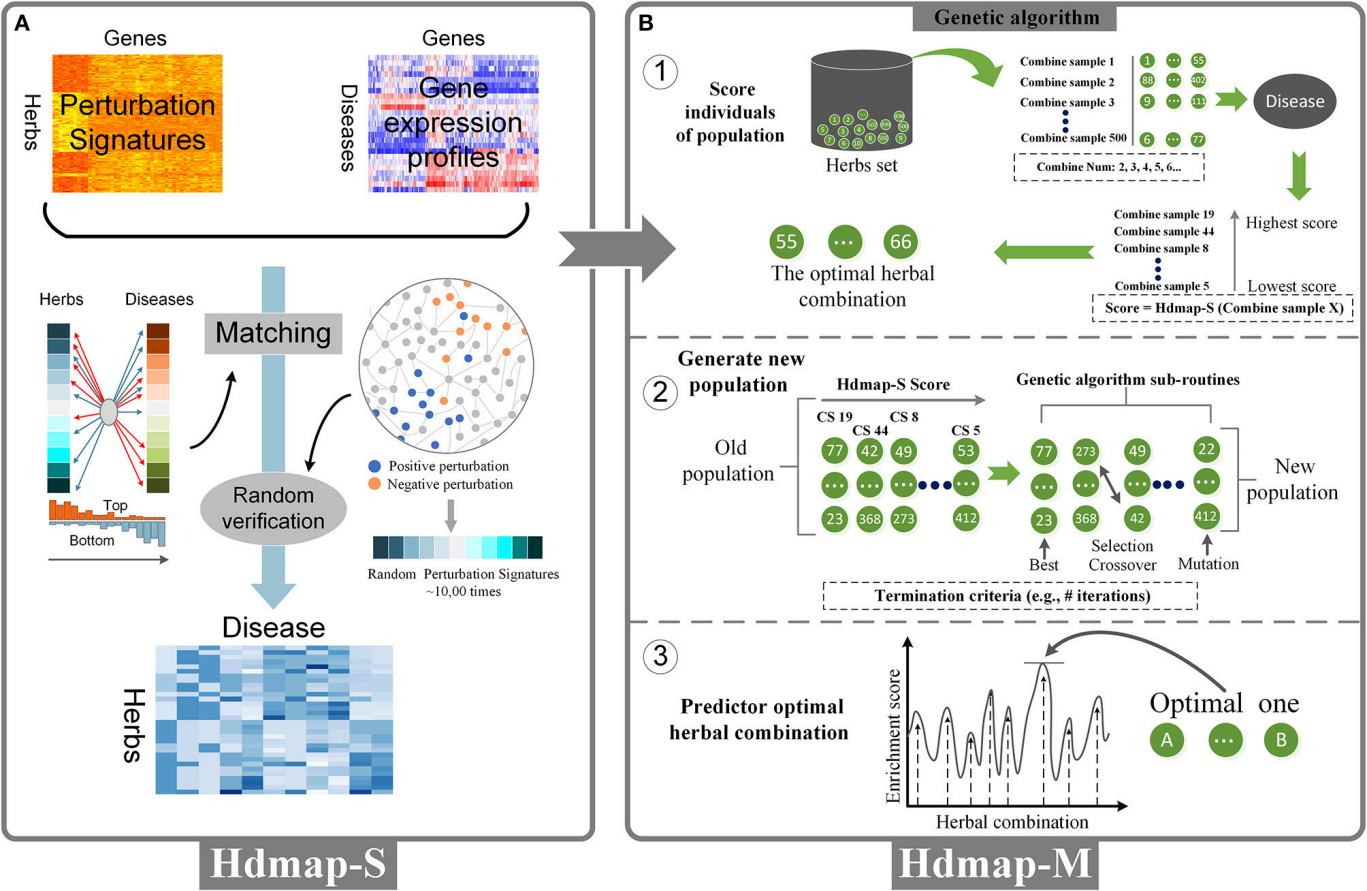
Overview of the HDmap-S and HDmap-M. **(A)** HDmap-S is used to query the herb-perturbation signatures against the disease reference expression set to assign an enrichment score to each herb-disease pair based on profile similarity. These scores are interpreted, resulting in a list of candidate therapeutics for each disease of interest. **(B)** HDmap-M is an HDmap-S-based genetic algorithm used to identify the optimal herbal combination. For each disease, the inputs of the algorithm are the disease-pathological state profile and all 502 herb-perturbation signatures; (1) At initiation, the genetic algorithm generates a “population” of a random combination of herbs, termed “individuals”, and assigns them the perturbation signatures. For each one, a matching score was calculated to evaluate the therapeutic potential of the herbal combination for the disease; (2) The genetic algorithm sub-routines are then used to generate a new population, biasing toward higher enrichment score. Optimal solutions are maintained without modification, and lower scoring individuals are combined (“crossed over”) and modified (“mutated”) to search the solution space in a heuristic manner. The termination criteria are typically the number of generations without improvement; (3) The genetic algorithm yielding the highest prediction enrichment score is considered the optimal herbal combination.

To predict an herb with potential therapeutic effects for the disease, we used the disease-pathological state profiles as a reference database and applied a system-wide interaction mapping strategy (HDmap-S, Single Herb-Disease Mapping) to query this database with all perturbation signatures of the herbs to generate a ranked list of the herbs for each disease. To this end, we calculated the relationship between each herb and disease as follows: (1) for each of the herbs, we determined an “optimal signature,” i.e., a subset of the extreme perturbation genes (the top and bottom genes) in the herb-perturbation signatures; (2) the HDmap-S score was calculated based on the matching degree between the perturbation signatures of the herbs at the top/bottom of the disease-pathological state profiles (Figure 2A).

Once the perturbation signatures had been obtained for each herb, we extracted the signatures {*h*_*top*_, *h*_*bottom*_} for each the herb, where *h*_*top*_ and *h*_*bottom*_ represented the top and bottom ranked genes, respectively. And this perturbation signatures were considered as a biological response to the treatment of the herb (Chang et al., 2003; Iorio et al., 2010). The size *t* of “optimal signature” was empirically determined to be 250, which the number of perturbation genes and the prediction performances of the algorithm were synthetically considered (Kidd et al., 2016).

To assess the matching degree between a pair of the herb and disease, the optimal signatures of the herb *h* were afforded as follow:

$$\begin{array}{l} h_{top}=\left\{h_{1}^{top},\ldots ,h_{t}^{top}\right\}\\ h_{bottom}=\left\{h_{1}^{bottom},\ldots ,h_{t}^{bottom}\right\} \end{array}$$

where *h*_*top*_ and *h*_*bottom*_ were the top/bottom-perturbation genes. Then we calculated a *Total Matching Score* (TMS), which represents HDmap-S score for each trio of herb, disease and gene set size (*h, d, t*), based on the overlap of the perturbation signatures of the herb at the top/bottom of the disease-pathological state profiles. We defined as follow:

$$TMS_{\left(h,d,t\right)}=\frac{MS_{d}^{h_{top}}-MS_{d}^{h_{bottom}}}{2}$$

where *d* is the reference disease pathological state profile, and $MS_{d}^{h_{top}/h_{bottom}}$ is the matching score of the perturbation signatures of the herb with respect to the disease *d*. And *TMS*_(*h, d, t*)_ ranges in [−1, 1], it is measure based on the methodology originally introduced by Lamb et al. (Lamb et al., 2006). If this measure is closed to 1 or −1, it means that the herb might be a possible treatment option for the disease, and vice versa.

The significance of a trio of herb, disease and gene set size (*h, d, t*) was estimated by the permutation test. The detailed information is shown as follows. For each (*h, d, t*), we calculated a total matching score *TMS*_0_. Then, we generated 10,000 random perturbation signatures and calculated the random total matching score *TMS*_*n*_, where *n* is an integer from 1 to 10,000. Finally, we calculated the *P*-value for each (*h, d, t*) as follows:

$$P_{\left(h,\;d,\;t\right)}=\left\{N/10,000|\sum_{i=0}^{n}\left|TMS_{i}\right|\geq \left|TMS_{0}\right|\right\}$$

where *N* is the number of times that |*TMS*_*i*_| ≥ |*TMS*_0_| is true, and the frequency of this event (*N*/10, 000) can be taken as a *P-value* (Supplementary Table 3).

### Prediction of an herbal combination for the disease (HDmap-M)

To predict an herbal combination with potential therapeutic effects for the disease, an HDmap-S-based genetic algorithm was implemented to identify the optimal herbal combination (HDmap-M, Multi-Herbs-Disease Mapping; Figure 2B). The matrix of perturbation intensity scores of all 502 herbs was input as well as the corresponding differentially expressed genes of one disease, which the locations of the genes were determined by the fold change. We first generated 500 candidate solutions (the herbal combination), in which the perturbation signatures of each solution were merged from the perturbation signatures of herbs in the solution. Besides, we excluded candidate solutions that violated the clinical herbal contraindications. Finally, all initial solutions were defined as *POP*_0_:

$$POP_{0}=\;\left[\begin{array}{c} c_{1}^{1}\;\cdots \;c_{1}^{m}\\ \vdots \;\ddots \;\vdots \\ c_{n}^{1}\;\cdots \;c_{n}^{m} \end{array}\right]$$

where *n* is the number of the solution and the number is 500, and *m* is the number of the perturbation signatures of the solution and is set to 500 (250 top and 250 bottom perturbation-genes) based on the above analysis, and $c_{i}^{j}$ represents the perturbation intensity of the *j-*th perturbation signature of the *i-*th solution. Then, each solution was scored to evaluate the treatment effect by the HDmap-S. These HDmap-S scores of all solutions were ranked and one solution with the largest absolute score was called as the optimal solution. The HDmap-S scores of all initial solutions were displayed as follow:

$$POP_{0}^{HDmap-S}=\left\{\left|TMS_{1}\right|,\left|TMS_{2}\right|,\ldots ,\left|TMS_{i}\right|,\ldots ,\left|TMS_{n}\right|\right\}$$

where |*TMS*_*i*_| is the HDmap-S score of the *i*-th solution. Subsequently, some solutions are selected through a score-based process, where a solution with high score is typically more likely to be selected to breed a new generation. And the selection method of the process was defined as follow:

$$P_{i}=\frac{\left|TMS_{i}\right|}{\sum_{j=1}^{n}\left|TMS_{j}\right|}$$

where *P*_*i*_ represents the probability that the *i*-th solution is selected. The next step is to generate a new generation population of solutions from those selected through a combination of genetic operators: crossover and mutation. And the parameters of genetic operators were based on the experiences from the previous research literature (Deb et al., 2002). The new generation population of solutions was defined as *POP*_1_:

$$POP_{1}=\;\left[\begin{array}{c} {c^{\prime }}_{1}^{1}\;\cdots \;{c^{\prime }}_{1}^{m}\\ \vdots \;\ddots \;\vdots \\ {c^{\prime }}_{n}^{1}\;\cdots \;{c^{\prime }}_{n}^{m} \end{array}\right]$$

The above generational process is repeated until the termination conditions have been reached. And the termination conditions are: (1) fixed number of generations reached; (2) The highest-ranking solution has been obtained, and successive iterations no longer produce better results. Finally, the optimal herbal combination for all disease cases was obtained according to the process described above. And we explored the differences between the herbal combinations and the classic TCM formulas. And we also further explore whether the herbal combinations obey to the role of “Jun-Chen-Zuo-Shi.” The classic TCM formulas with the information of “Jun-Chen-Zuo-Shi” were obtained from the Chinese Medicine Formula Image database (http://lib-nt2.hkbu.edu.hk/database/cmed/cmfid/index.asp?). And we compared the differences between the herbal combinations and the classic TCM formulas. The ingredients from the herbal combinations were considered as a set. And the ingredients of the classic TCM formulas were divided into four sets according to “Jun-Chen-Zuo-Shi”: the set of “Jun,” the set of “Chen,” the set of “Zuo,” and the set of “Shi.” And we counted the common number of ingredients between the herbal combinations and four sets of the classic TCM formulas, and the proportion of the four sets were also calculated. Finally, we chosen two examples to illustrate the differences between the herbal combinations and the classic TCM formulas (Supplementary Figures 3A–C). To further validate the reliability of the result calculated by the genetic algorithm, a published gene expression data of rheumatoid arthritis was used to predict the potential optimal herbal combination. And the treatment mechanism of combination for the rheumatoid arthritis is analyzed.

### Literature mining and verification

To count the utilization of herbs in the disease and validate our approach, we performed a large-scale text mining approach on the PubMed database. as follows. We first constructed the keyword of the herbs and diseases (Supplementary Table 1, 2). Then, we use the keywords to retrieve summaries of all PubMed articles from 1992 to 2017, to statistics the number of the keyword-related articles. And this process was done through an R package RISmed (Kovalchik, 2015), which could download content from NCBI databases. Finally, the hypergeometric distribution was applied to obtain the evaluate the significance of the co-occurrences of herb and disease:

$$P_{\left(h,\;d\right)}=1-\sum_{i=0}^{k-1}\frac{\left(\frac{K}{i}\right)\left(\frac{N-K}{n-i}\right)}{\left(\frac{N}{n}\right)}$$

where *N* is the total number of articles in PubMed (22,188,039 articles, as given by PubMed, access time: October 9, 2017), *K* is the number of the literatures associated with disease *d*, *n* is the quantity about herb *h*, *k* is the number of papers about the effects of corresponding herb *h* on disease *d*. *P*-value indicates the consequence of relevance between herb *h* and disease *d* (significant when *P*-value < 0.05).

### Gene ontology (GO) and pathway enrichment analysis

To determine the biological function of the novel herbal combination of disease, we enriched the overrepresented gene ontology (GO) terms and pathway terms of the perturbation signatures. For GO analysis, GO biological process (GOBP) terms were identified by DAVID database (https://david.ncifcrf.gov/; Huang et al., 2008a,b), which were represented the biological function influenced by the herbal combination. And GOBP terms with *P*-value < 0.01 and false discovery rate < 0.05 by Fisher's Exact test were observed. And the same screening conditions are used for pathway analysis.

### Network construction

To further explore the action mechanisms of the novel herbal combination in the treatment of rheumatoid arthritis, we constructed one network: Herb-“Perturbation Genes” network (H-PG network). In the network, the nodes represent herbs/targets, and edges represent they are linked with each other (Supplementary Table 6). Moreover, the edge width represents the perturbation intensity of the herb to target and the node size represents the perturbation intensity from the herbs. Besides, the targets are divided into three categories: targets were mainly perturbed by AR, targets were mainly perturbed by BR, and targets were mainly perturbed by RC. The degree of a node is the number of edges associated with it. The topological properties of these networks were analyzed using the Network Analysis plugin of Cytoscape (Shannon et al., 2003).

## Results

### Construction of a disease personalized treatment framework

To explore the synergistic mechanism of herbal medicine and the personalized treatment strategy for diseases, the establishment of standard basic data is the key factor for the algorithm. Hence, we first integrated the data of diseases and herbs on the molecular level including the transcriptional profiles of disease and the perturbation signatures of the herbs (see Methods). Finally, we obtained 460 disease-pathological state profiles (Figure 1B) including a series of differentially expressed genes, which represented the changes in gene expression from health to disease. And we also calculated 502 herb-perturbation signatures (Figure 1B), which represented the targets that affected by the ingredients in herb.

With the establishment of disease and herbal data, we perform a system-wide interaction mapping strategy to predict the most therapeutic potential herb for the disease based on the perturbation signatures, termed HDmap-S (Single Herb-Disease Mapping). The HDmap-S is a similarity-based modeling algorithm that computes a score for each pair of herbs and diseases by summarizing the herb-induced gene perturbation changes and transcriptional responses in disease states (Figure 2A). And the HDmap-S score for an herb defined the similarity of the expression pattern of the genes perturbed by the herbal combination to that of the differentially expressed genes in the disease state. This similarity-based approach was shown to be successful in predicting the interplay between drug targets and diseases (Sirota et al., 2011; Kidd et al., 2016). Specifically, in the present study, we assessed the similarity between herbs and diseases by comparing the perturbation profiles. To this end, we first extracted the perturbation signatures for each herb by selecting 250 genes at the top of the profiles (positive perturbation) and 250 genes at the bottom of the profiles (negative perturbation). Then we checked if the genes in the perturbation signatures ranked consistently at the top/bottom of the disease-pathological state profiles, and vice versa. Finally, an HDmap-S score of a pair of herb and disease was computed based on the overlap of the 250 top-ranked genes and bottom-ranked genes in each profile, and vice versa. And the higher HDmap-S score indicates a high match between herb and disease, which means that the herb is more likely to be used to treat this disease.

After determining the optimal single herb for the disease, we want to further identify the optimal herbal combination for the disease. However, it is a challenge to effectively determine the optimal herbal combination from a large number of candidate herbal combinations. To this end, we used a machine learning approach to select the optimal herbal combination of the disease based on the HDmap-S scores, termed HDmap-M (Multi-Herbs-Disease Mapping; Figure 2B). In brief, we first randomly generated 500 candidate herbal combinations and evaluated the association between these solutions and the corresponding disease. Then, these solutions underwent selection, crossover, and mutation to obtain new candidate herbal combinations and performed a reassessment. This process will continue to iterate until we found the optimal herbal combination for the disease. Obviously, the key point in this machine learning algorithm is how to evaluate the therapeutic potential of the herbal combinations for the disease. Hence, we analyzed the perturbation signatures of herbal combination and disease-pathological state profile with the HDmap-S to assess the therapeutic potential of herbal combinations for the disease. Finally, we constructed a disease personalized treatment framework.

### Model performance

#### The features of the perturbation signatures

**Figure 3:**
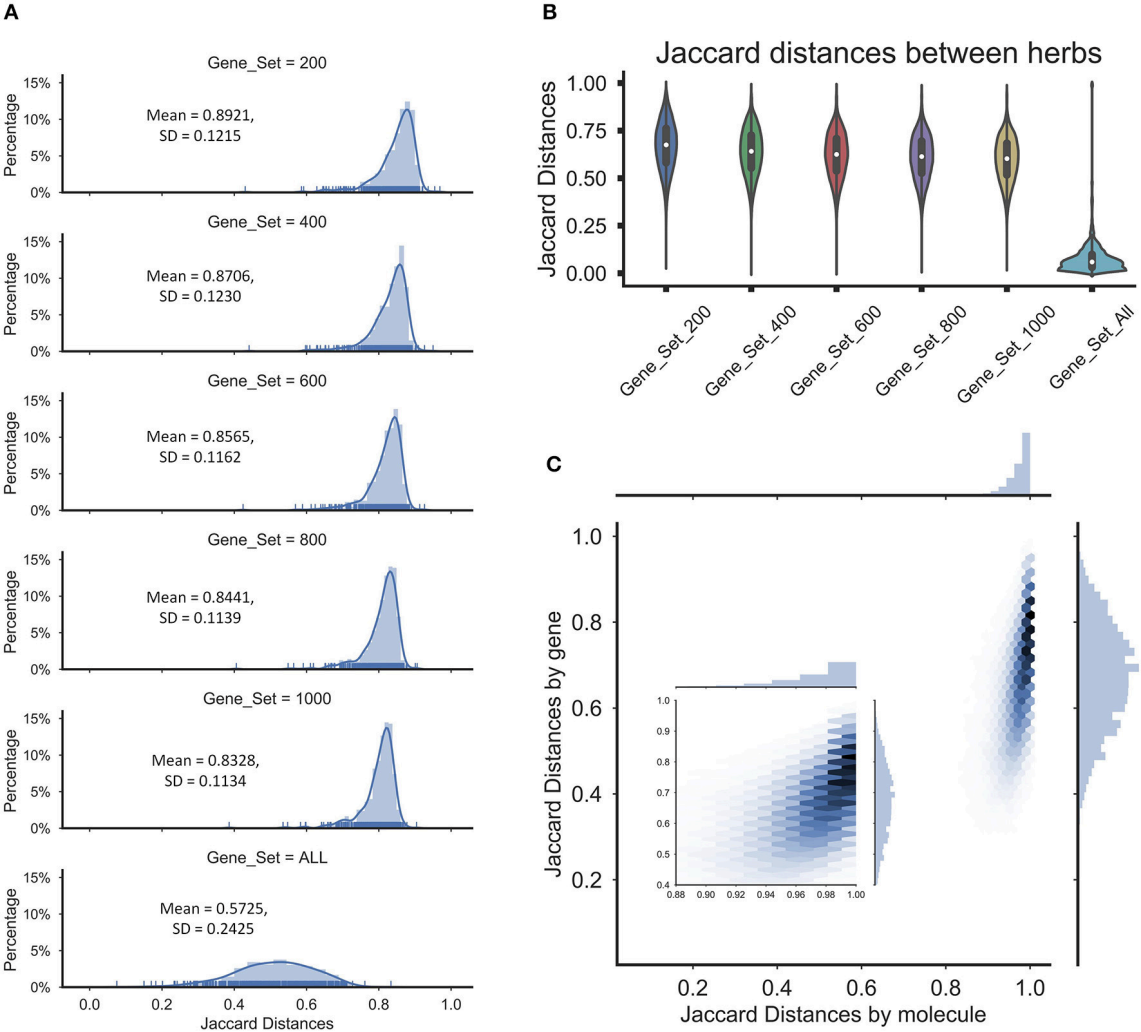
The Jaccard distances were calculated for the herbs and ingredients in herbs. **(A)** The distribution of the average Jaccard distances between molecules in herbs for different gene sets. **(B)** The violin diagram of Jaccard distances between the herbs for different gene sets. **(C)** The Hexbin plot with marginal distributions for the Jaccard distances based on the molecules or genes in herbs, which the gene set was 200.

To determine whether the operation will lead the final perturbation signatures of all herbs tended to be nearly equal, we assessed the overlapping among the perturbation signatures of herbs by the Jaccard distance between sets of the extreme perturbation genes (the top/bottom perturbation genes). First, we compared the distribution of the means of Jaccard distance between the perturbed genes of the ingredients in herbs to determine the similarity of the perturbations of the ingredients in the herbs, which the gene sets were including 200 perturbation genes, 400 perturbation genes, 600 perturbation genes, 800 perturbation genes, 1,000 perturbation genes, and all perturbation genes. And we found that the average Jaccard distance of extreme disturbance genes between ingredients in herbs exceeded overall average background distance (Figure 3A), which suggested that the different perturbation genes of the ingredients in the herbs may make the herbal perturbation signatures to be diversity. Furthermore, nearly 10,000 genes were perturbed by the herbs and the Jaccard distance between herbs based on all the perturbed genes was small, indicating that the herbs perturbed the entire network and the perturbations tended to be uniform (Figure 3B). However, despite the high coincidence of the perturbation signatures of herbs, the average Jaccard distance of extreme disturbance genes showed significant differences between the herbs (Figure 3B), and these differences are not affected by repetitive molecular between herbs (Figure 3C). These data indicate that each herb has a unique therapeutic mechanism by perturbing various target groups, while partial overlap of herbal target groups also implied the possibility of combination therapy of herbs.

#### The performance of the HDmap-S

To evaluate the performance of the HDmap-S, we use herbs as an example to predict their relationship with diseases based on the HDmap-S. Finally, we calculated the HDmap-S score for 94,878 pairs of herbs and diseases. To further evaluate the significance of the predicted herb-disease interactions, we used a permutation approach under which random perturbation signatures were generated and the analysis was repeated 10,000 times for each pair of herb and disease (see Methods). We computed the *P-*value of individual herb-disease score values and the complete computational integration of the profiles of the herbs and diseases produced 15,035 potential connections between herbs and diseases (*P*-value < 0.05). And each of the 502 herbs was averagely significantly associated with at least 29 diseases (Supplementary Table 3). Subsequently, we verified these potential connections between herbs and diseases through the literature from PubMed. In brief, we first investigated the PubMed by a large-scale text mining with the keywords of herbs and diseases. Then, the hypergeometric distribution was applied to obtain the chance improbable of co-occurrences of each herb and disease. Finally, we verified the accuracy of HDmap-S through the relationship of herbs and diseases that we found in the literature. And we found that the HDmap-S model performs well in predicting the interactions of disease and herbs with the accuracy of 83.25%. Overall, these results serve to highlight the fact that the application of HDmap-S can provide more information and solutions for the treatment of diseases.

#### The properties of HDmap-S on herbs

**Figure 4:**
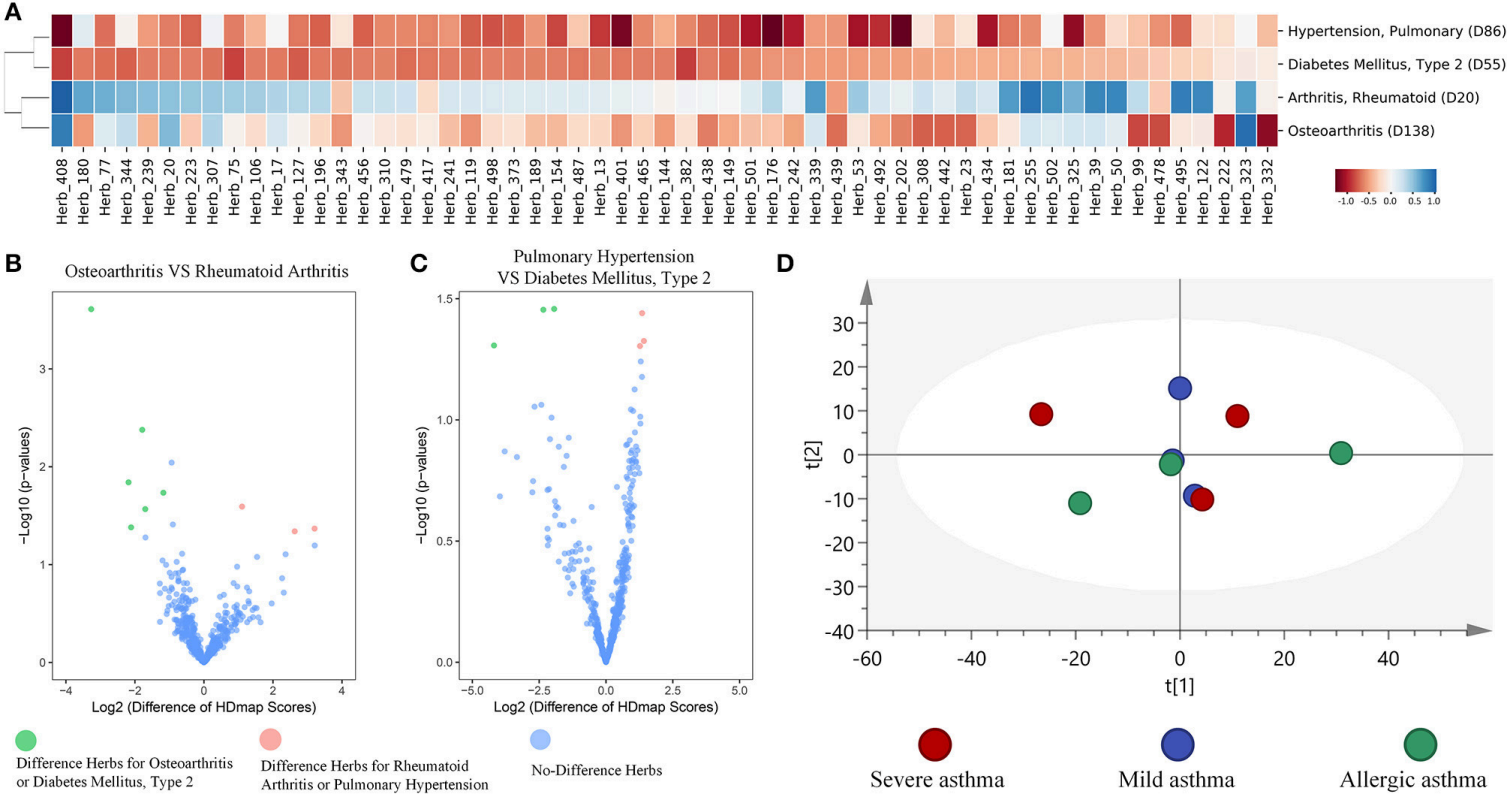
Global properties of HDmap-S. **(A)** Heat map of disease-herb scores, which included four diseases [Arthritis, Rheumatoid (D20), Osteoarthritis (D138), Hypertension, Pulmonary (D86) and Diabetes Mellitus, Type 2 (D55)] and 57 herbs (the merger of the best top 15 herbs for each disease). Red indicates a negative (therapeutic) disease-herb score. Blue indicates a positive disease-herb score. **(B)** A volcano plot of herbs for osteoarthritis and rheumatoid arthritis. Green indicates the different herbs for osteoarthritis across to rheumatoid arthritis, and pink indicates the different herbs for rheumatoid arthritis across to osteoarthritis. **(C)** A volcano plot of herbs between pulmonary hypertension and type 2 diabetes mellitus. Green indicates the different herbs for type 2 diabetes mellitus across to pulmonary hypertension, and pink indicates the different herbs for pulmonary hypertension across to type 2 diabetes mellitus. **(D)** Principle component analysis of the Hdmap-S scores for six asthma pathological state profiles that mapped to mild asthma, severe asthma, and allergic asthma. Red indicates severe asthma, blue indicates mild asthma, and green indicates allergic asthma. It is seen that these three states of asthma were overlap and difference.

To further examine the global landscape of the HDmap-S, we used the HDmap-S score as a similarity metric and organized the complete set of herb and disease interactions through unsupervised hierarchical clustering. We then looked separately at the clustering of diseases based on their similarity scores across herbs (Supplementary Figure 2). And we found that diseases tended to be co-occurrence or had similar pathogenesis were clustered together, which suggested that similar treatment strategy was applied to these diseases (Figure 4A). For example, the Arthritis, Rheumatoid (D20) and Osteoarthritis (D138), which revealed similar pathogenesis including inflammation, pain, cartilage damage and so on (Pap and Korb-Pap, 2015), formed a cluster. Besides, the Hypertension, Pulmonary (D86) and Diabetes Mellitus, Type 2 (D55) that overlap in the population (Tsimihodimos et al., 2018) were also clustered together. And despite the clustering of these two diseases, we also found the differences in the matching herbs between the two diseases based on the result of the volcano plot, and the same situation existed within rheumatoid arthritis and osteoarthritis (Figures 4B,C). Even more, we further found that the different manifestations of the same disease correspond to different herbs. For example, six asthma pathological state profiles correspond to three states that including mild asthma, severe asthma, and allergic asthma, were used to compute the matching scores for each herb. Reassuringly, principal component analysis (PCA) of the HDmap-S scores showed that different states of asthma were partial overlap, which suggested that the therapeutic strategies for different states of asthma were not only similar but also *expand*ed (Figure 4D). These results indicate that the HDmap-S could provide disease-specific personalized treatment strategies.

#### The properties of the HDmap-M

**Figure 5:**
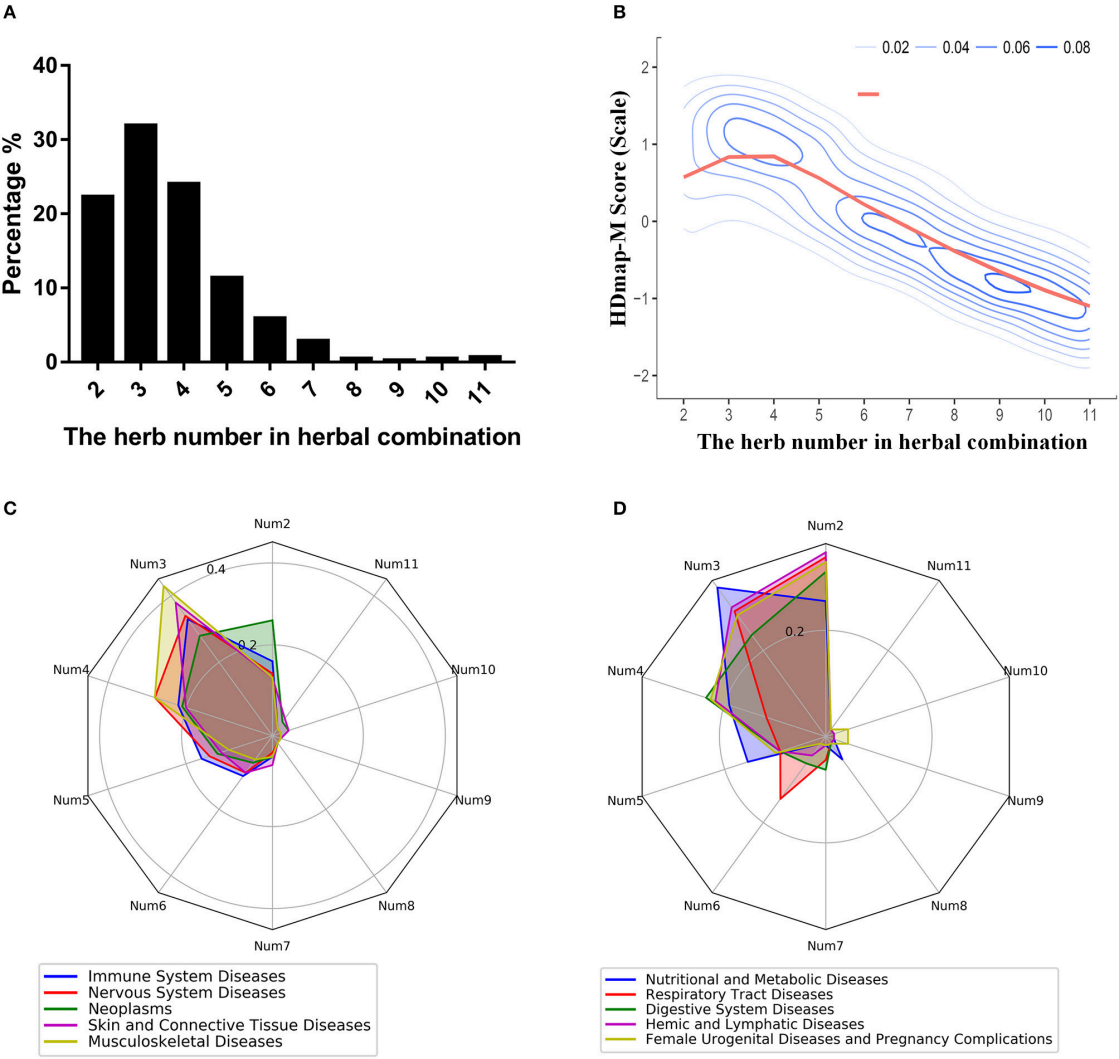
The properties of the HDmap-M. **(A)** The distribution of the number of herbal combinations of all disease cases. **(B)** The density plot for the HDmap-M scores of a different number of the herbal combination. **(C,D)** The equiangular spokes radar chart. Each spoke describes the number of the herbal combination. The length of a spoke is the percentage of the disease category for this number of the herbal combination. And different colors indicate different disease classifications.

To explore the global landscape of the disease personalized treatment framework, we analyzed the characteristics of all herbal combinations for the 189 diseases based on the framework. And we found that the number of the herbs in herbal combination mainly concentrated in the range from 2 to 5, which occupied 89.52% of all disease cases (Figure 5A). At the same time, we also observed such a trend of the matching scores of the herbal combination that it peaked when the number of combinations was 3 and then gradually declined (Figure 5B). Then, we sought to determine whether the number of herbal combinations for different categories of diseases was differences. We found that diseases with the characteristic of complex pathogenesis and multiple inducement factors were required more herbs for combination therapy, like the neoplasms, nervous system diseases, digestive system diseases and so on (Figures 5C,D). These results suggested that the efficacy of herbal combination did not increase with the increase of the number of herbs, and on the contrary, the number of herbal combinations that achieved the best therapeutic effect was located within a limited area. Furthermore, we also explored the differences between the herbal combinations and the classic TCM formulas. Interestingly, we found that some herbal combinations are consistent with the classic TCM formulas. For instance, we have predicted an herbal combination for breast neoplasm (DDED0205): *Arum Ternatum Thunb*. (Herb_35) and *Zingiber officinale Roscoe* (Herb_367) (Supplementary Figure 3A). And this herbal combination is same with the Minor Pinellia Decoction, which is a classic TCM formula that has been reported to have potential therapeutic effects on the side-effects of chemotherapy in breast cancer patients (Zhang et al., 2007). At the same time, we found that some herbal combinations are similar to the classic TCM formulas. For example, we predicted an herbal combination for Crohn disease (DDED0059): *Rhizoma Cimicifugae* (Herb_366), *Radix Puerariae* (Herb_499), and *Dioscoreae Hypoglaucae Rhizoma* (Herb_124) (Supplementary Figure 3B). Interestingly, we found that this herbal combination is similar to the Cimicifuga and Pueraria Decoction and obeyed to the role of “Jun-Chen-Zuo-Shi.” The GO analysis and pathway analysis indicated that the targets of herbal combination mainly influenced the steroid hormone biosynthesis (hsa00140, *P*-value < 0.01) and steroid hormone mediated signaling pathway (GO:0043401, *P*-value < 0.01; Supplementary Figure 3C). And the steroid hormone is the main treatment for Crohn disease (Andus et al., 2003). These results indicated that HDmap-M could provide novel TCM formula for the diseases.

### Prediction of the novel formula of the rheumatoid arthritis

Rheumatoid arthritis (RA) is considered a higher occurrence autoimmune inflammatory disease that mainly targets the synovial membrane, cartilage, and bone, and is caused by a series of risk factors including the genetic susceptibility, sex, and age, smoking, infectious agents, hormonal, dietary, socioeconomic, and ethnic factors (Alamanos and Drosos, 2005; Mcinnes and Schett, 2007; Singh et al., 2016). This disease is associated primarily with articular inflammation, synovial joint damage and increasing disability over time, which is increasingly recognized to promote the transmission of cardiovascular morbidity, psychological impairment, the risk of cancer and osteoporosis (Firestein, 2014; Mcinnes et al., 2016). Although biologic therapy and targeted therapy have enabled good therapeutic successes, the remission rates, particularly off-therapy, remain low, and re-establishment of immune homeostasis is elusive for all but a minority (Smolen and Aletaha, 2015; Catrina et al., 2016). Fortunately, Traditional Chinese Medicine (TCM), especially herbal medicine that has been in use for more than 3,000 years, can provide a more flexible approach to obtain various combinations and compatibility of herbs according to the specific physiological conditions of diseases and patients. And, rheumatoid arthritis has been clinically treated with specific herbs or herbal combinations for many years. In the following sections, we determined a novel formula for the rheumatoid arthritis based on the HDmap-M: *Boschniakia Rossica* (BR, Herb_53), Rhizoma *Cimicifugae* (RC, Herb_366), *Arisaematis* Rhizoma (AR, Herb_394). Multiple studies have reported the therapeutic effects of herbs from this predicted formula on rheumatoid arthritis (Supplementary Table 4). And results of the previous study demonstrated that BR-extract exerted significant anti-inflammatory activities, macrophage activation effect, and antioxidative activities in both chronic and acute inflammation process (Tadashi et al., 1994; Yin et al., 2000; Liu et al., 2011). And RC, known as a natural product to treat pain and inflammation, was reported to increase the proliferation of stem cell and increase the osteogenic differentiation of stem cells (Kim and Kim, 2000; Lee et al., 2017). Furthermore, published literature documented that AR has anti-inflammatory activity and is one of the commonly used herbs for the management of painful osteoarthritis (Wang et al., 2012; Sun et al., 2017). Taken together, these results suggested that the combination of these three herbs has a potential therapeutic effect against rheumatoid arthritis.

**Figure 6:**
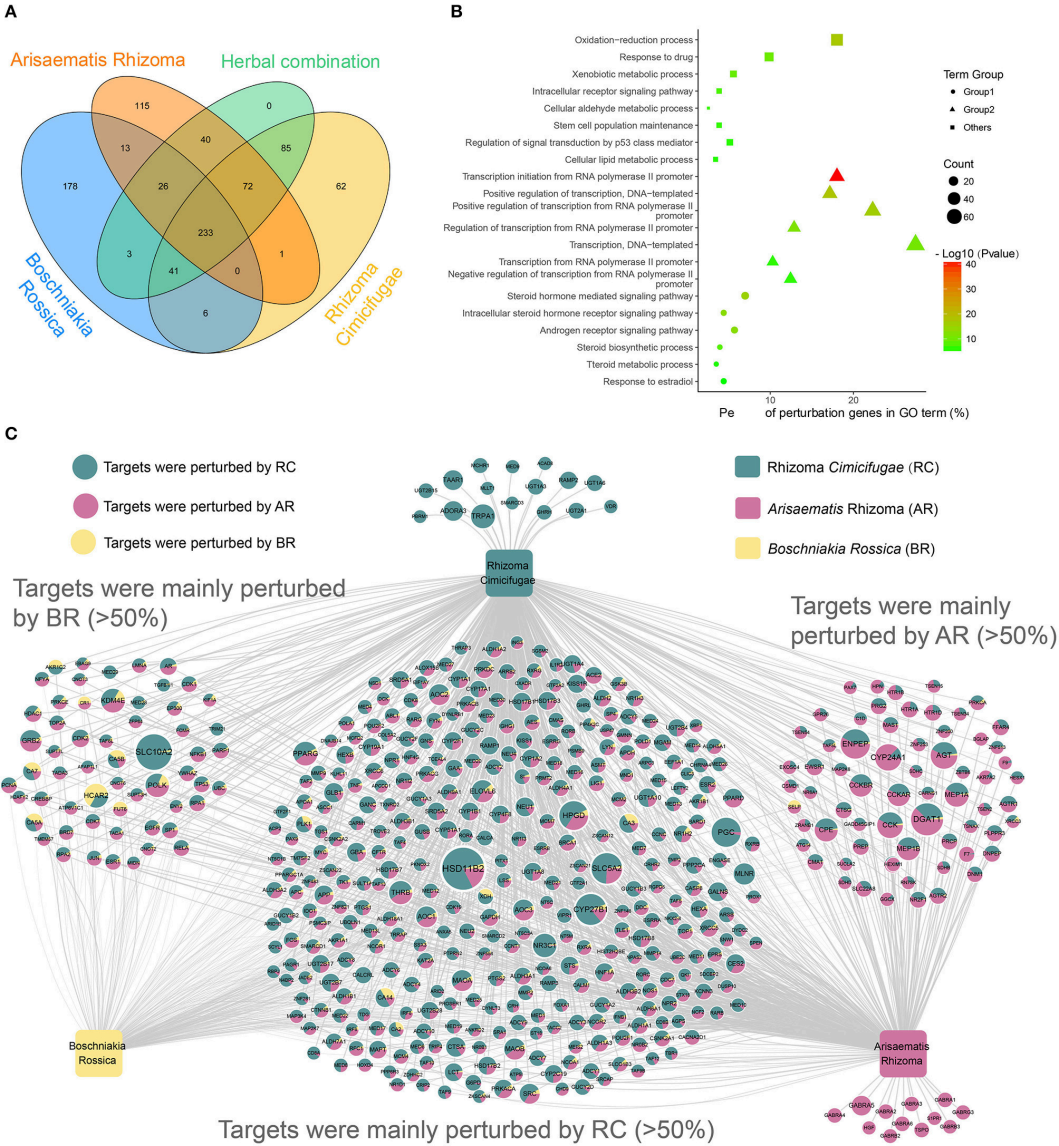
Overview of the novel formula. **(A)** Four-set Venn diagram analysis of the perturbation genes by three herbs and their combination. **(B)** Gene Ontology (GO) analysis of the common perturbation genes of the three herbs in the herbal combination. The y-axis shows significantly enriched “Biological Process” categories in GO of the target genes, and the x-axis shows the percent of perturbation genes in GO term (%). And the shapes represent three categories: the functions of transcriptional and translational regulation (triangle), the functions in metabolic process and signaling pathway (rounded), and other functions (square). Besides, the size of the shapes represents the number of the perturbation genes and the color of the shapes represents the enrichment scores of these terms (*P*-value < 0.01). **(C)** The Herb-Perturbation Genes network (H-PG network) of the herbal combination. The herbal combination includes three herbs: *Boschniakia Rossica* (yellow), Rhizoma *Cimicifugae* (Turquoise.), *Arisaematis* Rhizoma (pink). There are 500 genes nodes. The size of the gene nodes represents the perturbation intensity that affected by the herbal combination. And the ratio of the three colors on the nodes represents the contribution of the respective herbs to the perturbation intensity.

To assess the therapeutic mechanism of the herbal combination and the interaction among the herbs, a Venn diagram (Figure 6A) has been used to quantify the distribution of BR, RC, AR and the herbal combination in the perturbation genes. Among the 500 perturbing genes of the herbal combination, we found that the three herbs co-perturbed 233 genes (Figure 6A), suggesting that the related biological processes of the genes were suffered from strong perturbation by the herbs to improve the symptoms of rheumatoid arthritis. To further explore the function of these genes, we performed GO enrichment analysis on the 233 genes. Finally, 21 GOBP terms were considered as the most relevant function of the herbal combination (Supplementary Table 5). And the GO enrichment results provided the corresponding evidence that the herbs achieved therapeutic effects by perturbing rheumatoid arthritis (RA)-related biological processes (Figure 6B). The common gene set of three herbs showed GO enrichment for the synthesis, metabolism and signal regulation of steroid hormone (Figure 6B-rounded), a class of endogenous substances that regulated the immune system and inflammation and was considered as an important substance involved in the treatment of rheumatoid arthritis (Grossman, 1985; Buttgereit et al., 2011; Coutinho and Chapman, 2011). And the herbs may regulate steroid hormone through their influence on the transcription process (Figure 6B-triangle). Furthermore, this herbal combination also associated with the oxidation-reduction process, the stem cell population maintenance, drug metabolism process and regulation of signal transduction by the p53 class mediator, which were required for effective therapeutic intervention of RA (Phillips et al., 2010). Overall, these results indicate that the herbal combination treats RA from multiple levels of anti-inflammatory effects, immunosuppressive effects, drug metabolism and so on, through regulation of endogenous steroid hormones and modulation of intracellular signaling.

To decipher the action mechanism of herbal combination and discover the most potential RA-linked key targets, we generate the Herb-Perturbation Genes network (H-PG network). The bipartite H-PG network graph (Figure 6C; Supplementary Table 6) was constructed for the 500 perturbation genes by connecting to the three herbs through 1,360 interactions. Fascinatingly, we found that the perturbation strong for the herbs on the same genes was differentiated and each herb has a gene set that contributed more than 50% of the perturbation strong, particularly the RC and AR. For example, HSD11B2 [Corticosteroid 11-beta-dehydrogenase isozyme 2] is the target mainly perturbed by RC and is involved in the activation of synthetic glucocorticoids and modulation of intracellular glucocorticoid levels, that is also one of the main drugs to treat RA (Orsida et al., 2002; Mullins et al., 2015). And in the present studies, the HCAR2 (Hydroxycarboxylic acid receptor 2), perturbed by BR, demonstrated anti-inflammatory effects by activates a downstream pathway mediated through activation of the AMPK/SIRT1 axis, inhibiting the transcription factor NF-κB, and subsequently the secretion of pro-inflammatory cytokines (Kieseier and Wiendl, 2015). Moreover, AR could also alter the lipid levels by influencing the DGAT1 (Diacylglycerol O-acyltransferase 1) to reduce the inflammatory burden and cardiovascular risk in RA patients (Koliwad et al., 2010). These results suggested that each herb in the herbal combination has its unique treatment mechanism for RA.

## Discussion

The mapping of herbs to diseases is of great importance in the clinical treatment of traditional medicine. The prevailing approach to obtain the herbal combination is based on established tools and techniques developed for screening libraries of drugs with optimal pharmacodynamics and pharmacokinetics, which is a labor-intensive and costly process. Computational approaches are naturally suited to overcome the high costs and other logistical limitations associated with the experiment, allowing for capturing the optimal herbal combination for the disease. And it is worth noting that most of these approaches are applicable only to the well-characterized information (e.g., when the ingredients of the herb are documented). Fortunately, with the rapid accumulation of genomics in the past decade, expression profile-based methods do not require any prior information about the object, which are the most general ones (Musa et al., 2017). For example, the “Connectivity Map” (Lamb et al., 2006; Lamb, 2007), one of the most promising approaches, is a method based on “gene signatures.” This method has been used to clarify multiple biological issues, such as interaction analysis between drugs and immune cell types (Kidd et al., 2016), inferring host response to infection (Han et al., 2015), etc. And we have gained tremendous inspiration from this method to establish a connection between herbs and diseases. Unfortunately, the herbs face severe challenges and suffer from an insufficient accumulation of genomics (Wang et al., 2011; Zhang et al., 2012), which makes it difficult to obtain perturbation data of herbs. Hence, the existence of reliable and valid profiles to assess the herbs are essential to bridge the current gap between diseases and herbs.

Systems pharmacology and network perturbation approach developed in recent years may be able to provide such a framework to solve this problem owing to the huge chemical informatics data (e.g., the connections between the compounds and targets) that obtained in the past decade (Barabasi et al., 2011; Cheng et al., 2012; Huang et al., 2014; Woo et al., 2015). And protein-protein interaction (PPI) network, as the basic data of network perturbation approaches, has been derived in attempts to shed light on the exogenous substances underlying influence, which had successfully applied to elucidate the treatment mechanisms of drugs and new drug discovery (Menche et al., 2015; Guney et al., 2016). Therefore, the integration and application of these methods may be an appropriate starting point to reveal the accurate and consistent gene perturbation signatures in biological systems imparted by the herbs. And we could provide a way to compensate the defects in the herbal data, which could establish the links between herbs and diseases.

In the present study, we describe the integrative computational approach to map the effects of herbs on diseases. And this approach exhibits reasonable reliability based on the results of random verification and literature verification. These results indicate that this reliable computational approach would have a role in the development of traditional medicine. In addition, attention needs to be particularly paid to the basic profiles in the construction of the algorithm. Although a large amount of biochemistry information about the herbs has been accumulated over the past decade, there is still some herb's biochemical information is not complete. Hence, the refinement of this algorithm is depended on the identification of the ingredients of herb and the determination of ingredient direct targets through the research of the pharmacologist and chemist. Fortunately, the development of chemical technology and biological experiment technology has made it possible to solve this problem and improve the generalization ability of our algorithm in predicting the relationship between herbs and diseases.

Moreover, the novel herbal combination predicted by our algorithm confirmed that our method is reliable and can provide a reference for the personalized clinical treatment of rheumatoid arthritis. And the global trends extracted from our data could provide guidelines and specific predictions on how to treat the disease, uncover a complete picture of the complexity of herb effects on the disease, and identify the new therapeutic target. And the unknown interactions identified in the method may include a lot of information that warrant experimental follow-up. Overall, our results validate the concept of computational analysis of public gene expression databases as a potentially useful approach to the clinical treatment of herbs that may uncover additional uses for herbs, which will help us better understand their treatment mechanism.

## Author contributions

DG, AL, and YW provided the concept and designed the study. XC, CZ, and CW conducted the analyses and wrote the manuscript. XC, CZ, and CW made an equal contribution to the work. ZG, SG, ZN, CH, CL, and YF participated in data analysis. DG, AL, and YW contributed to revising and proof-reading the manuscript. All authors read and approved the final manuscript.

### Conflict of interest statement

The authors declare that the research was conducted in the absence of any commercial or financial relationships that could be construed as a potential conflict of interest.

## Supplementary material

Supplementary Figure 1 Herb-perturbation signatures generation framework. **(A)** Generate the initial “Perturbation signatures” for the herbs and ingredients based on the databases and approves that including TCMSP, ChEMBL, Binding DB, WES, and PreAM. **(B)** Generate the final “Perturbation signatures” of the herbs and ingredients based on the thermal diffusion.

Supplementary Figure 2 The Heat map of the HDmap-S scores for the herbs and diseases.

Supplementary Figure 3 Comparison of classic TCM formulas and herbal combinations. **(A)** The herbal combination for breast neoplasm (DDED0205) is same with the Minor Pinellia Decoction. **(B)** The herbal combination for Crohn disease (DDED0059) is similar to the Cimicifuga and Pueraria Decoction. The composition of the ingredients between the herbal combination and Cimicifuga and *Pueraria* Decoction illustrates that these two formulas have same “Jun” drugs and “Chen” drugs, and the “Zuo” drugs and “Shi” drugs showed a certain degree of overlap. **(C)** The analysis of the therapeutic mechanism of the herbal combination: Rhizoma *Cimicifugae*, Radix *Puerariae*, and Dioscoreae Hypoglaucae Rhizoma.
