# Supplementary figures and images for: Systems-Mapping of Herbal Effects on Complex Diseases Using the Network-Perturbation Signatures
Source: Front Pharmacol. 2018 Oct 18;9:1174. doi: 10.3389/fphar.2018.01174 (PMC6201628; doi:10.3389/fphar.2018.01174)

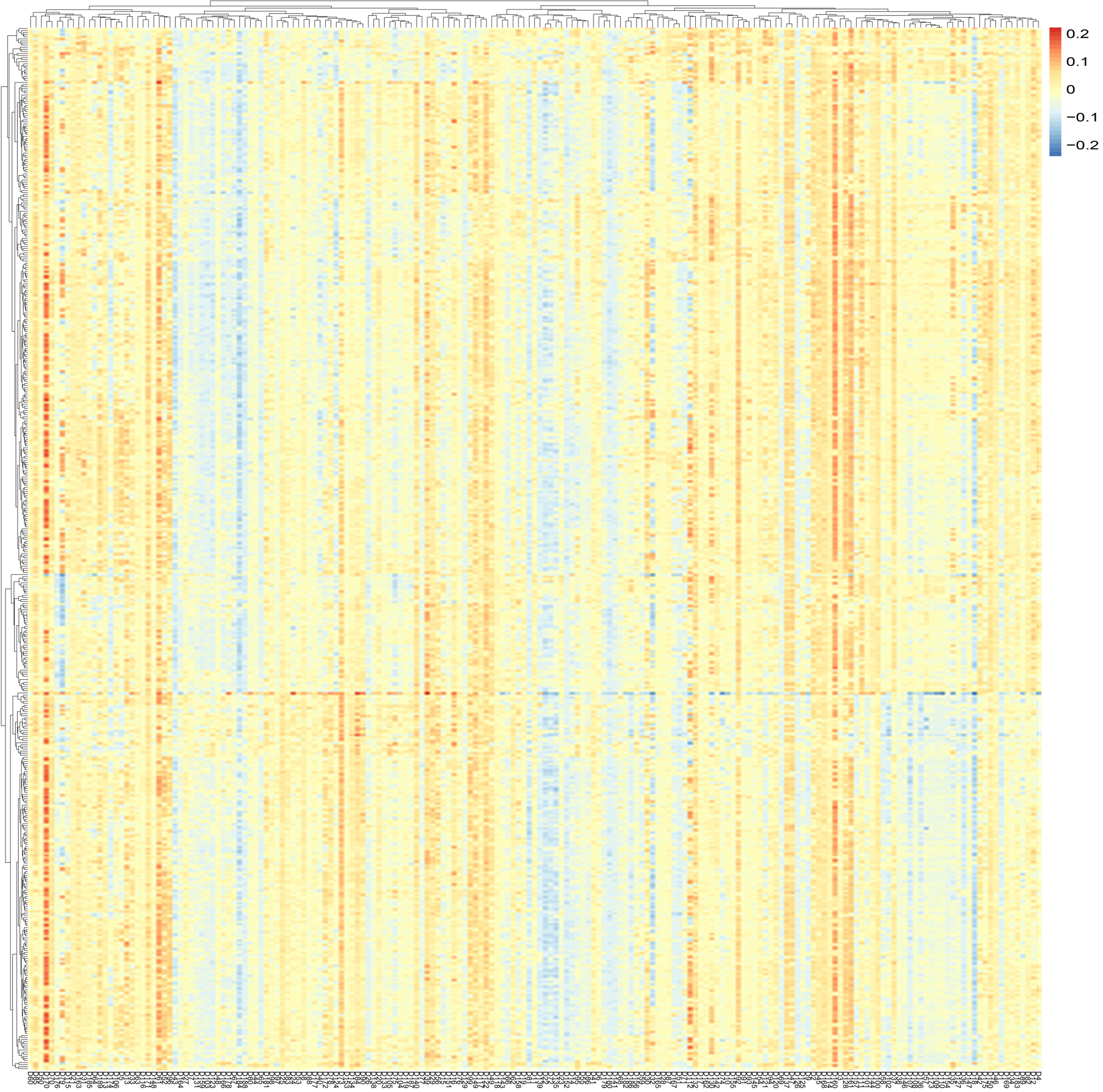

Supplement: Supplementary Figure 1 — Herb-perturbation signatures generation framework. (A) Generate the initial “Perturbation signatures” for the herbs and ingredients based on the databases and approves that including TCMSP, ChEMBL, Binding DB, WES, and PreAM. (B) Generate the final “Perturbation signatures” of the herbs and ingredients based on the thermal diffusion. [file Data_Sheet_1.ZIP › Supplementary Figure 2.tif]

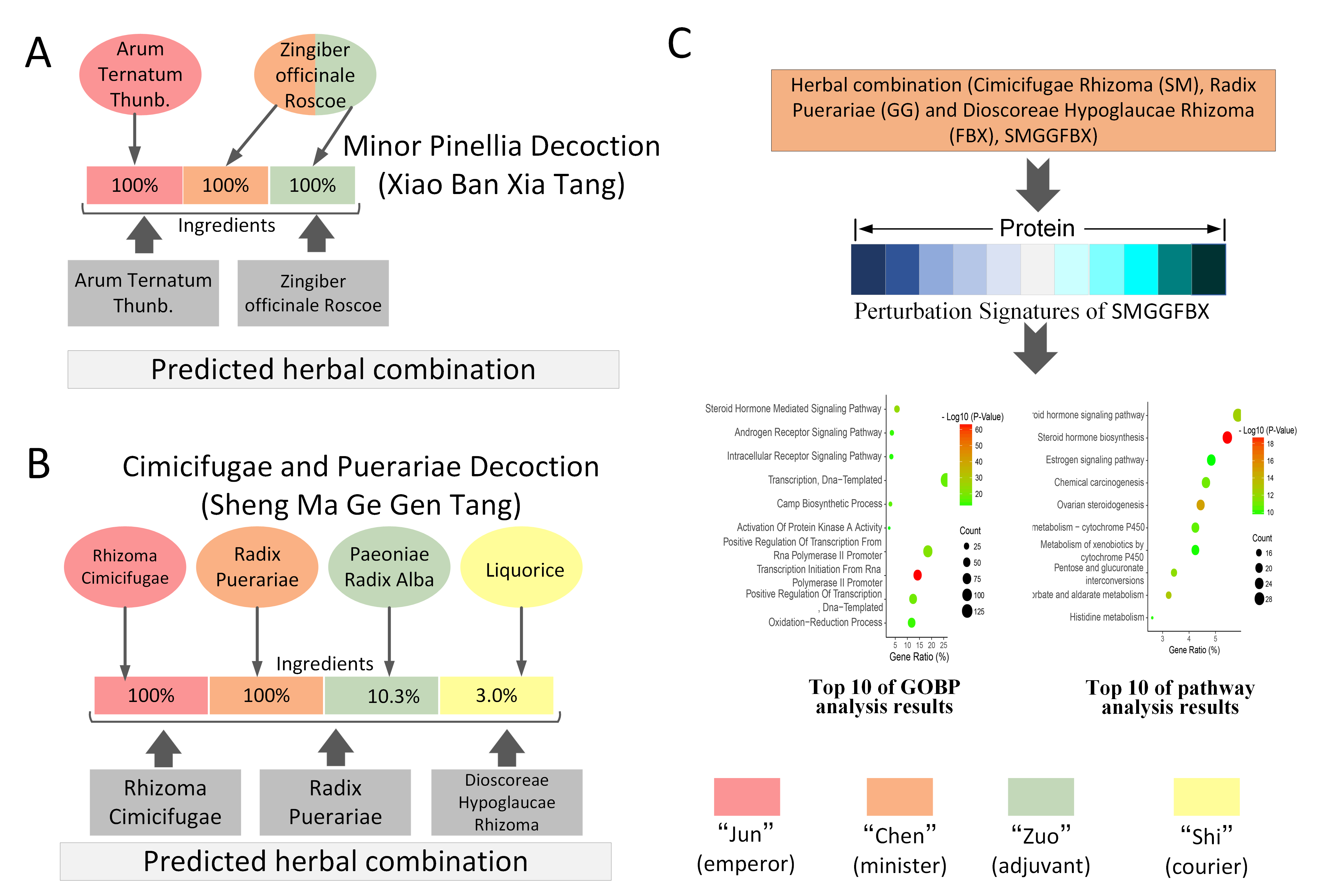

Supplement: Supplementary Figure 1 — Herb-perturbation signatures generation framework. (A) Generate the initial “Perturbation signatures” for the herbs and ingredients based on the databases and approves that including TCMSP, ChEMBL, Binding DB, WES, and PreAM. (B) Generate the final “Perturbation signatures” of the herbs and ingredients based on the thermal diffusion. [file Data_Sheet_1.ZIP › Supplementary Figure 3.tif]

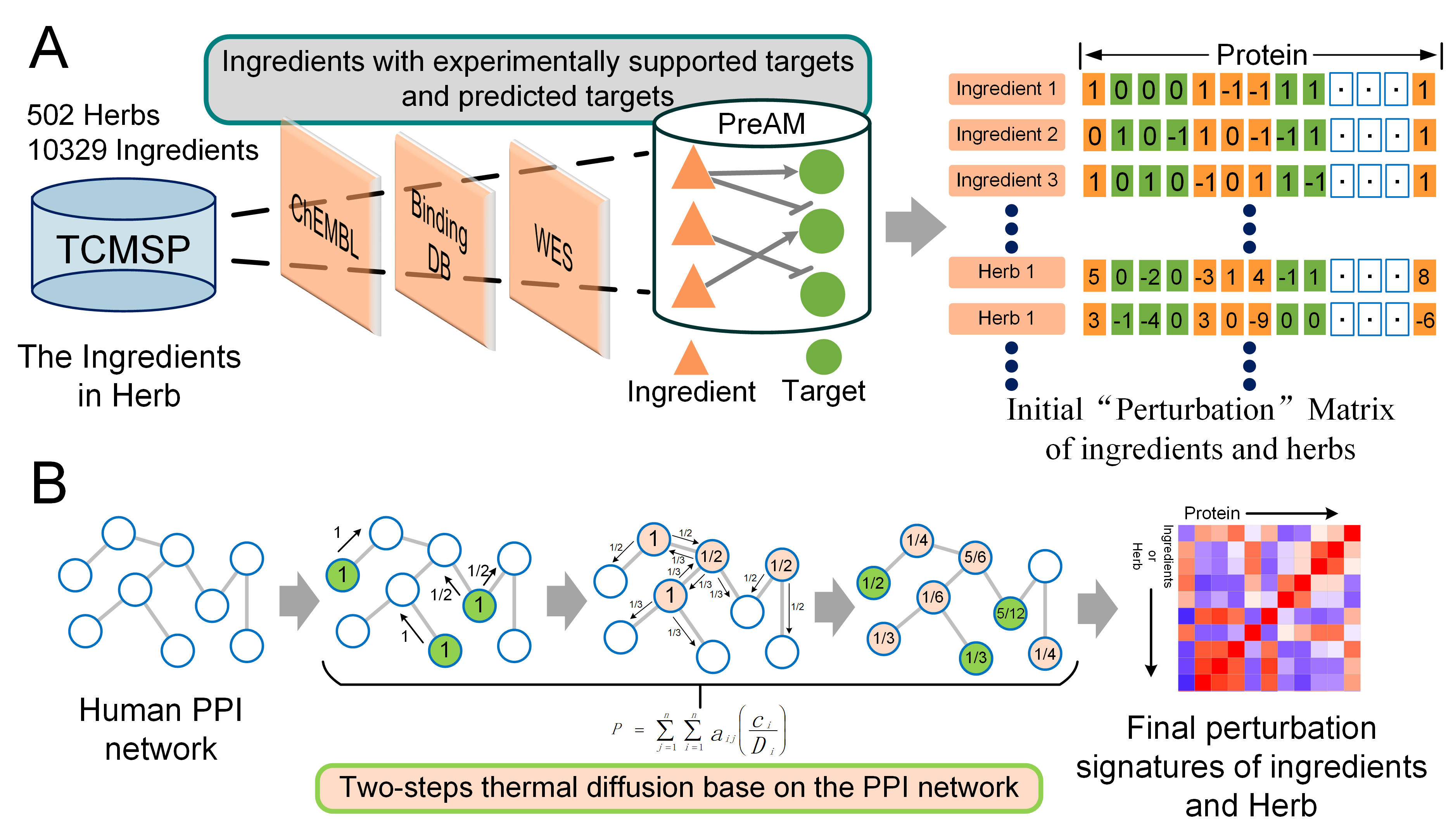

Supplement: Supplementary Figure 1 — Herb-perturbation signatures generation framework. (A) Generate the initial “Perturbation signatures” for the herbs and ingredients based on the databases and approves that including TCMSP, ChEMBL, Binding DB, WES, and PreAM. (B) Generate the final “Perturbation signatures” of the herbs and ingredients based on the thermal diffusion. [file Data_Sheet_1.ZIP › Supplementary Figure 1.tif]
